# Predicting superagers: a machine learning approach utilizing gut microbiome features

**DOI:** 10.3389/fnagi.2024.1444998

**Published:** 2024-09-09

**Authors:** Ha Eun Kim, Bori R. Kim, Sang Hi Hong, Seung Yeon Song, Jee Hyang Jeong, Geon Ha Kim

**Affiliations:** ^1^Department of Artificial Intelligence Convergence, Ewha Womans University, College of Artificial Intelligence, Seoul, Republic of Korea; ^2^Ewha Medical Research Institute, Ewha Womans University, Seoul, Republic of Korea; ^3^GI&Neurology Part, R&D, CJ Bioscience, Seoul, Republic of Korea; ^4^Oncology Part, R&D, CJ Bioscience, Seoul, Republic of Korea; ^5^Department of Neurology, Ewha Womans University Mokdong Hospital, Ewha Womans University, College of Medicine, Seoul, Republic of Korea

**Keywords:** gut microbiome, superagers, machine learning, LightGBM algorithm, Alistipes

## Abstract

**Objective:**

Cognitive decline is often considered an inevitable aspect of aging; however, recent research has identified a subset of older adults known as “superagers” who maintain cognitive abilities comparable to those of younger individuals. Investigating the neurobiological characteristics associated with superior cognitive function in superagers is essential for understanding “successful aging.” Evidence suggests that the gut microbiome plays a key role in brain function, forming a bidirectional communication network known as the microbiome-gut-brain axis. Alterations in the gut microbiome have been linked to cognitive aging markers such as oxidative stress and inflammation. This study aims to investigate the unique patterns of the gut microbiome in superagers and to develop machine learning-based predictive models to differentiate superagers from typical agers.

**Methods:**

We recruited 161 cognitively unimpaired, community-dwelling volunteers aged 60 years or from dementia prevention centers in Seoul, South Korea. After applying inclusion and exclusion criteria, 115 participants were included in the study. Following the removal of microbiome data outliers, 102 participants, comprising 57 superagers and 45 typical agers, were finally analyzed. Superagers were defined based on memory performance at or above average normative values of middle-aged adults. Gut microbiome data were collected from stool samples, and microbial DNA was extracted and sequenced. Relative abundances of bacterial genera were used as features for model development. We employed the LightGBM algorithm to build predictive models and utilized SHAP analysis for feature importance and interpretability.

**Results:**

The predictive model achieved an AUC of 0.832 and accuracy of 0.764 in the training dataset, and an AUC of 0.861 and accuracy of 0.762 in the test dataset. Significant microbiome features for distinguishing superagers included Alistipes, PAC001137_g, PAC001138_g, Leuconostoc, and PAC001115_g. SHAP analysis revealed that higher abundances of certain genera, such as PAC001138_g and PAC001115_g, positively influenced the likelihood of being classified as superagers.

**Conclusion:**

Our findings demonstrate the machine learning-based predictive models using gut-microbiome features can differentiate superagers from typical agers with a reasonable performance.

## 1 Introduction

While cognitive decline is traditionally viewed as an inevitable feature that occurs with aging (Hedden and Gabrieli, 2004), recent research has identified a subset of older adults known as “superagers” (Rogalski et al., 2013; Sun et al., 2016). These individuals maintain cognitive abilities comparable to those of middle aged adults (Harrison et al., 2012; Gefen et al., 2015) or young adults (Harrison et al., 2018; Zhang et al., 2020). Since cognitive health has consistently been regarded as an important factor for quality of life of older adults (Reichstadt et al., 2007), investigating the neurobiological characteristics associated with superior cognitive function in superagers is essential for understanding “successful aging” (Depp and Jeste, 2006).

A number of evidence suggests that gut microbiome plays a key role in brain function (Galland, 2014; Mohajeri et al., 2018). The brain, gut, and gut microbiome form a bidirectional communication network known as the microbiome-gut-brain axis (Martin et al., 2018). Previous research has indicated a link between alterations in the gut microbiome and the increased oxidative stress and inflammation, which are biological markers of cognitive aging (Komanduri et al., 2019). Changes in gut microbiome composition have been associated with neurocognitive disorders; for example, dementia is linked to microbiome alterations along with elevated biomarkers indicating increased gut permeability and inflammation. Specifically, the Lachnospiraceae NK4A136 group, a potential producer of butyrate, is found at reduced levels in individuals with dementia (Stadlbauer et al., 2020). This highlights the potential impact of gut microbiota on cognitive health.

The importance of gut microbiota in cognitive function is further supported by studies indicating that the gut microbiome can influence the brain through multiple pathways, including the production of neuroactive compounds, modulation of systemic inflammation, and maintenance of gut barrier integrity (Galland, 2014). These mechanisms suggest that a healthy and balanced gut microbiome may contribute to the preservation of cognitive function and resilience against age-related cognitive decline.

Despite these insights, the specific characteristics of the gut microbiome that contribute to superior cognitive function in superagers remain underexplored. Understanding these characteristics could provide new avenues for promoting cognitive health in aging populations.

This study, therefore, aims to investigate the unique patterns of the gut microbiome in superagers and to develop machine learning-based predictive models that can differentiate superagers from typical agers based on individual gut microbiome features with reasonably high performance. Additionally, we aim to validate the model through various perspectives, including SHAP (SHapley Additive exPlanations) analysis and correlation analysis between model predictions and cognitive scores.

## 2 Materials and methods

### 2.1 Participants

Community-dwelling volunteers aged 60 years or older were recruited from the Gangseo or Yangcheon Center for Dementia, one of the public facilities for dementia prevention in Seoul. A total of 161 older adults agreed to participate in this study. A neurologist evaluated eligibility using the following inclusion criteria: aged 60 years or older, able to read and write, scored > − 1.5 SD of the mean of age and education-matched norm on the Korean version of Mini-Mental State Examination, 2nd edition (K-MMSE-2) (Baek et al., 2016) and with normal cognitive function defined as scoring higher than-1 SD (16th percentile) of the demographically matched norm on the tests of memory, attention, language, visuospatial, and frontal executive functions in the Seoul Neuropsychological Screening Battery-II (SNSB-II) (Ryu and Yang, 2023). We excluded individuals with any of the following characteristics: (1) suspected or diagnosed with mild cognitive impairment or dementia; (2) suspected or diagnosed major neurological or psychiatric illnesses, including major depressive disorders; (3) structural abnormalities that can affect cognitive functions on brain magnetic resonance imaging (MRI); (4) visual or hearing impairments severe enough to interfere with questionnaire response; (5) a history of medications that could affect cognitive and emotional functions in the last 3 months; or (6) any other major medical problems such as cancer.

**Figure 1:**
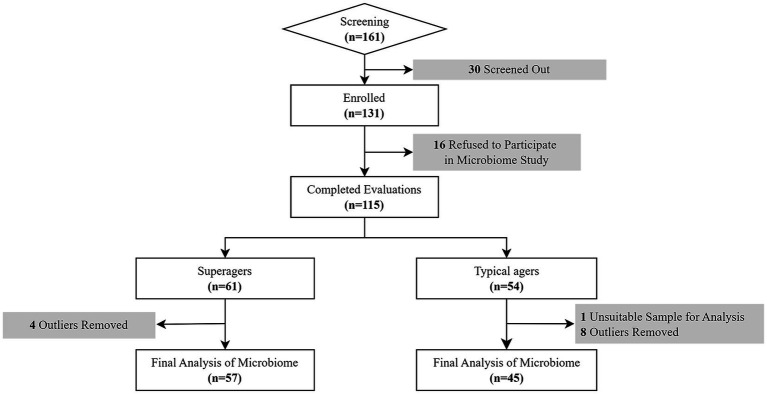
Flow chart of the study.

Of those 161 participants, 30 individuals did not meet the inclusion criteria while 16 refused the evaluation of the study including microbiome study. Therefore, a total of 115 older adults finally participated in this study (Figure 1).

The definition of superagers was based on their memory performance at or above average normative values of middle-aged adults (45 years old) on tests of delayed recall in both the Seoul Verbal Learning Test (SVLT) and the Rey Osterrieth Complex Figure Test (RCFT) and whose scores in other cognitive domains such as attention, language, visuospaital and frontal executive functions were at least average for age (Harrison et al., 2012; Sun et al., 2016; Bott et al., 2017; Dang et al., 2019). Based on these criteria, among 115 participants, there were 61 superagers and 54 typical agers.

In this study, we collected data from participants that included cognitive scores from neuropsychological assessments, gut microbiome profiles, demographic information, BMI (Body Mass Index), and dietary intake data from questionnaires. The gut microbiome data was primarily used to develop classification models for identifying superagers, while the demographic characteristics, cognitive scores, BMI, and dietary intake information were used to examine the characteristics associated with the superagers and gut microbiome.

Written informed consent was obtained from all participants prior to study participation, and this study was approved by the Institutional Review Board of Ewha Womans University Mokdong Hospital (IRB approval number: 2020–11–004-017).

### 2.2 Neuropsychological assessments

All participants were administered a standardized neuropsychological battery called the SNSB-II (Ryu and Yang, 2023): Digit Span Test (DST) forward and backward for attention; the Korean version of the Boston Naming Test (K-BNT) for language; the RCFT for visuospatial function and visual memory; the SVLT for verbal memory; and phonemic Controlled Oral Word Association Test (COWAT), Korean-Color Word Stroop Test (K-CWST) for executive functions. Age-and education-specific z-scores for each cognitive domain were used for the current study.

### 2.3 Gut microbiome data acquisition and preprocessing

#### 2.3.1 Stool sample collection and DNA extraction

Microbiome data were collected from the fecal samples of the participants. Out of the 115 participants, one sample from a typical ager was not available for analysis. Consequently, fecal samples from 114 participants were analyzed. To maximize microbial cell lysis for DNA extraction, the stool samples were homogenized by shaking in a sterile screw cap tube containing zirconia beads (2.3 mm, 0.1 mm diameter) and glass beads (0.5 mm diameter) using FastPrep-24 (MP Biomedicals, Santa Ana, CA, USA) for 50 s. After lysis, genomic DNAs from the homogenized stool samples were extracted using the Qiagen DNA Stool Mini Kit (Qiagen, Germantown, MD, USA) according to the manufacturer’s protocols.

#### 2.3.2 16S rRNA gene sequencing and taxonomic profiling

The V3-4 hypervariable region of the 16S rRNA gene was amplified with primers 341F and 805R using the direct polymerase chain reaction method. Libraries were prepared using a NEBNext Ultra II FS DNA Library Prep Kit for Illumina (New England Biolabs, Ipswich, MA). The prepared DNA libraries were sequenced by CJ Bioscience Inc. using the Illumina MiSeq platform (Illumina, San Diego, CA) with 2 × 300 base pair (bp) kit.

The DNA samples remaining after library construction were stored in a deep freezer at −60°C. The paired-end raw 16S rRNA sequences data were uploaded to EzBioCloud and processed using a web-based EzBioCloud microbiome taxonomic profile tool.^1^ High-quality sequence reads were assigned to “species group” at 97% sequence similarity using the PKSSU4.0 database.

### 2.4 Diet and nutritional intake questionnaire

We collected information on dietary habits and nutritional intake that might affect composition of gut microbiome, using the Computer Aided Nutritional Analysis Program CAN-Pro 5.0 (The Korean Nutrition Society, Seoul, Korea). It is designed to calculate personal nutrient intake and food consumption based on the Dietary Reference Intakes for Koreans 2015 (Welfare and Society, 2015). It assesses 108 nutrients, including 39 fatty acids and 21 amino acids, by evaluating the amounts of food consumption. The questionnaire includes 3,926 foods and 1,784 dishes and employs the 24-h recall method to obtain responses from each subject.

### 2.5 Development of classification models

#### 2.5.1 Feature selection

Considering the inter-individual variation in microbiome counts, we utilized the relative abundances (%) of each bacterial taxonomy as features in our models. To mitigate bias arising from skewed data, we excluded bacteria with a significant number of missing values at the phylum level, specifically those missing in more than half of the participants. This approach allowed us to focus on specific phyla—Firmicutes, Bacteroidetes, Proteobacteria, and Actinobacteria—along with their genera. For outlier identification, we employed the Tukey’s fences method at the phylum level. We defined outliers as values outside the range of Q1-1.5 times the interquartile range (IQR) and Q3 + 1.5 times the IQR.

Following this procedure, microbiome data from 12 participants (8 typical agers and 4 superagers) were excluded from the original group of 114. Consequently, the final analysis included microbiome data from 102 participants, consisting of 57 superagers and 45 typical agers, as shown in Figure 1.

After removing outliers, four phyla remained: Firmicutes, Bacteroidetes, Proteobacteria, and Actinobacteria. These phyla were the most abundant, constituting over 90% of all identified phyla (Supplementary Figure 2A). Within these phyla, we included their genera as features for our model. We selected 67 genera from Firmicutes, 3 genera from Bacteroidetes, 9 genera from Proteobacteria, and 4 genera from Actinobacteria. This resulted in a total of 83 features initially selected after outlier removal.

We employed Recursive Feature Elimination with Cross-Validation (RFECV) to analyze this dataset of 83 features. RFECV evaluates scores generated by different combinations of features, iteratively removing those with low importance, and ultimately identifies the optimal feature set through cross-validation. Consequently, 8 features were selected for developing the models.

Among the Firmicutes phylum, the selected features included the genus Leuconostoc from the family Leuconostocaceae, as well as genera from Clostridia such as PAC001115_g, PAC000194_g, PAC001137_g, PAC001138_g, PAC001236_g, and Romboutsia. For the Bacteroidetes phylum, the selected features included the genus Alistipes. There were no selected features from the Actinobacteria or Proteobacteria phyla.

#### 2.5.2 Model development

Machine learning algorithms, specifically utilizing the boosting-based ensemble model LightGBM, were employed to develop classification models for categorizing superagers. To ensure objective assessment of model performance, 20% of the data was set aside as a test set.

Additionally, 4-fold cross-validation was conducted on the training data to validate the model’s performance and optimize parameters. A random search with 50 iterations per search was performed. Subsequently, the models with high training performance were identified. Among them, the final model was selected based on test performance, indicating its ability to generalize well. Further enhancing model performance, manual threshold adjustments were made with a step size of 0.01.

To estimate feature importance, we used the ‘gain’ method, which sums the reduction in loss for splits where the feature is used across all trees. This total gain indicates how much the feature improves model performance.

The models were developed using Python 3.10 (Python Software Foundation, Delaware, United States) and the LightGBM 4.0.0 package (Microsoft Corporation, Washington, United States) along with scikit-learn 1.2.2 (Pedregosa et al., 2011).

#### 2.5.3 Assessment of model performance

The classification model’s performance was evaluated using several key metrics: accuracy, sensitivity, and the Area Under the Curve (AUC) of the Receiver Operating Characteristic (ROC) curve. Accuracy measures the ratio of correct predictions to total predictions, providing an overall indication of the model’s precision. Sensitivity, also known as the True Positive Rate (TPR), assesses how well the model identifies ‘superagers’ by measuring the ratio of correctly identified superagers to all actual superagers. Additionally, the AUC of the ROC curve was examined. AUC represents the area under this curve, with values ranging from 0.5 for a random classifier to 1 for a perfect classifier.

These performance metrics were employed to evaluate the model through 4-fold cross-validation and to assess predictions on test dataset. We compared model performance based on feature selection and algorithms, identifying the superior models. Performance outcomes were presented using performance tables and ROC curves.

The performance table also includes additional metrics such as precision, specificity, and F1 scores. Precision measures the proportion of instances classified as superagers that are genuinely superagers. Specificity assesses the ratio of instances correctly classified as typical agers among all actual typical agers. The F1 score, the harmonic mean of precision and recall (sensitivity), provides a balanced measure of a model’s performance. These metrics together provide a comprehensive evaluation of the model’s effectiveness in distinguishing between superagers and typical agers.

#### 2.5.4 Shapley additive explanations (SHAP)

The feature importance derived from the model provides insights into the magnitude of their impact but lacks the ability to explain the decision-making processes. To gain a deeper understanding of these mechanisms, we employed Explainable AI (XAI), a technology that facilitates the explanation and interpretation of the decision-making processes of machine learning and artificial intelligence models.

SHAP is an XAI technique that explains how each feature influences predictions by calculating Shapley values. These values, originating from cooperative game theory, quantify a feature’s contribution by assessing its impact across all possible feature combinations, contingent upon the inclusion or exclusion of the specific feature. SHAP simplifies the model’s complexity into a linear approximation, thereby elucidating the model’s behavior.

Utilizing SHAP enables us to scrutinize the impact of each feature on the model’s predictions, elucidating both the degree and direction of influence. For instance, it aids in understanding how features contribute to categorizing individuals as ‘superagers.’ Positive Shapley values indicate an increase in prediction values, while negative values indicate a decrease. SHAP 0.45.0 (Lundberg and Lee, 2017) was utilized in our analysis.

### 2.6 Statistical analyses

Before conducting each statistical analysis, a Shapiro–Wilk test was performed to assess the normality of the data distribution. Since the data did not follow a normal distribution, nonparametric tests were employed.

Statistical analyses were conducted to examine participant characteristics. Mann–Whitney U-tests were used to identify differences between typical agers and superagers in terms of demographic characteristics, cognitive performance, nutrient intake, and microbiome composition.

Following model construction, correlation analyses were performed to examine the relationships between class probabilities and cognitive scores. All analyses were conducted at a significance level of *p* < 0.05 using Python with SciPy 1.10.1 and statistics packages (Virtanen et al., 2020).

## 3 Results

### 3.1 Characteristics of participants

Demographic characteristics and cognitive performance *z*-scores are presented in Table 1. There were no significant differences in age, education, and BMI between superagers and typical agers. Similarly, no significant differences were observed in nutritional intake between the two groups. For both alpha diversity and beta diversity, no differences were shown between superagers and typical agers (Supplementary Figure 1). As expected, superagers demonstrated superior performance in memory, visuospatial, language, and frontal executive functions compared to typical agers.

**Table 1:** Demographics of superagers and typical agers.

	Superagers (*N* = 57)	Typical agers (*N* = 45)	*p*-value
*N* = 102	Mean (SD)	Mean (SD)	
Demographic characteristics			
Age (years)	73.26 (5.58)	72.47 (6.47)	0.580
Education (years)	11.16 (4.17)	11.00 (4.02)	0.748
Female, n (%)	45 (78.95)	34 (75.56)	0.689
BMI (kg/m^2^)	24.47 (3.45)	24.88 (3.25)	0.349
K-MMSE 2	28.75 (1.39)	28.42 (1.49)	0.234
Nutrient intakes			
Energy (kcal)	2130.96 (1008.60)	2186.57 (1115.63)	0.952
Carbohydrates (g)	325.54 (154.21)	335.63 (166.23)	0.866
Protein (g)	79.84 (41.24)	82.42 (47.33)	0.984
Fat (g)	56.71 (32.69)	56.11 (36.70)	0.618
Fiber (g)	35.66 (18.47)	35.85 (19.73)	0.845
Calcium (mg)	801.79 (379.89)	836.57 (443.64)	0.731
Magnesium (mg)	138.40 (82.01)	135.50 (84.11)	0.609
Sodium (mg)	4549.01 (2819.25)	4598.65 (3028.57)	0.835
Potassium (mg)	4032.18 (2172.25)	4157.64 (2410.67)	0.909
Cognitive performance (*Z*-scores)			
Digit Span Forward Backward	−0.04 (0.92)	−0.09 (0.91)	0.469
K-BNT	0.49 (0.76)	0.12 (0.74)	0.015*
RCFT copy	0.42 (0.57)	0.03 (0.72)	0.003*
SVLT delayed recall	1.12 (0.69)	0.21 (1.02)	< 0.001*
RCFT delayed recall	0.98 (0.88)	−0.13 (1.02)	< 0.001*
COWAT phonemic	1.20 (1.41)	0.57 (1.13)	0.012*
StroopTest color reading	0.79 (0.73)	0.41 (0.88)	0.028*

Data are shown as mean (SD, standard deviation) or number (%). BMI, Body Mass Index; K-MMSE, Korean version of the Mini-Mental State Examination; CDR, Clinical Dementia Rating; BNT, Boston Naming Test; RCFT, Rey Complex Figure Test; SVLT, Seoul Verbal Learning Test; COWAT, Controlled Oral Word Association Test.

*p < 0.05, the *p* value was obtained by Mann–Whitney U test.

### 3.2 Classifying models predicting superagers based on microbiome characteristics

**Figure 2:**
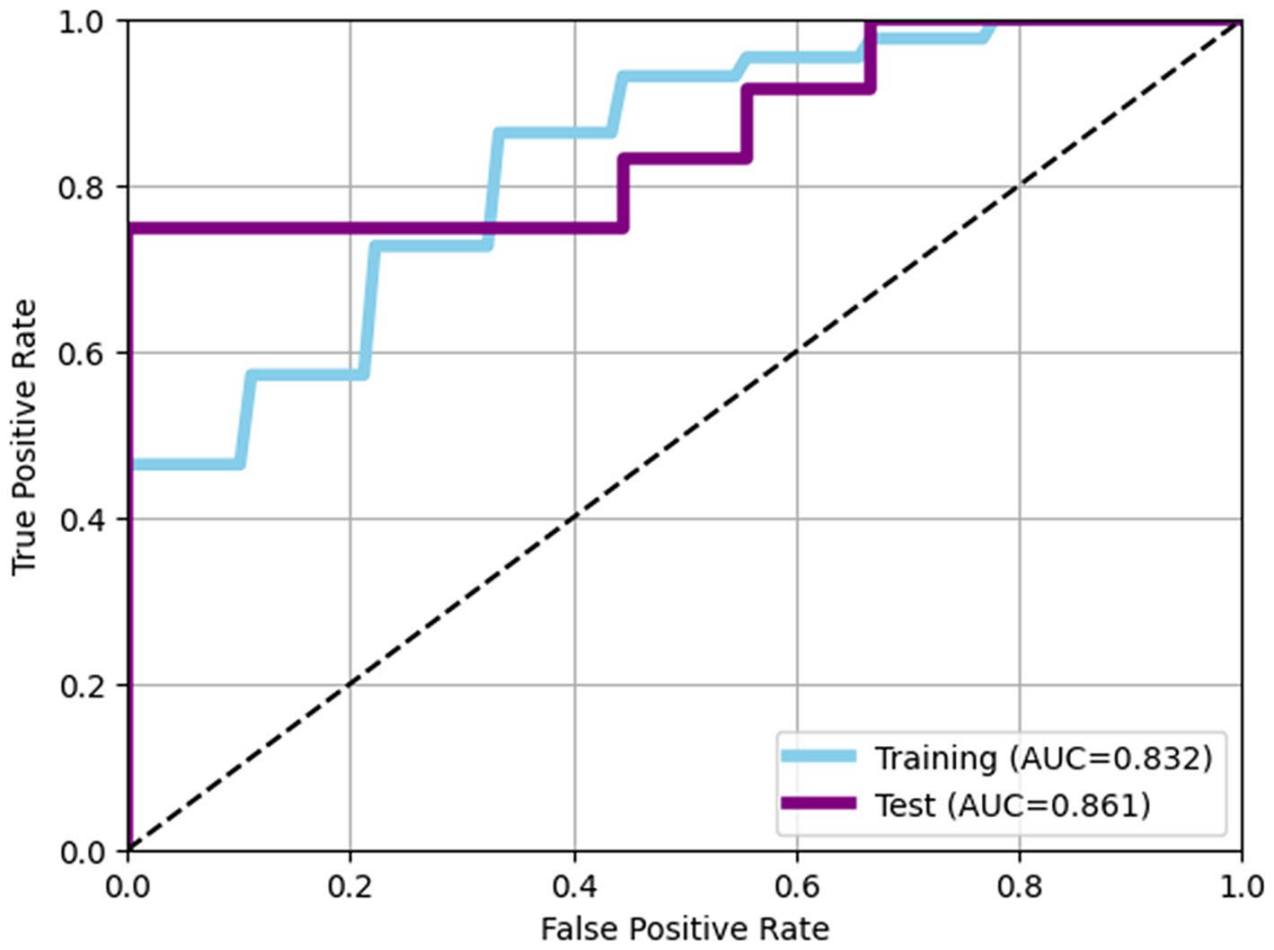
Receiver operating characteristic (ROC) curves of models. In the training data set, the model achieved an AUC of 0.832 with an accuracy of 0.764, while in the test dataset, the model achieved an AUC of 0.861 with accuracy of 0.762.

The performance of predictive model was evaluated using LightGBM algorithm, incorporating eight bacterial features selecting through Recursive Feature Elimination with Cross-Validation (RFECV). In the training data set, the model achieved an AUC of 0.832 with an accuracy of 0.764, while in the test dataset, the model achieved an AUC of 0.861 with accuracy of 0.762 (Table 2; Figure 2).

**Table 2:** Performance of the classification model for superagers.

Model	AUC	Accuracy	Precision	F1	Sensitivity	Specificity
4-fold cross validation of the training dataset (*n* = 81)						
LGBM	0.832	0.764	0.806	0.782	0.773	0.750
Test dataset (*n* = 21)						
LGBM	0.861	0.762	0.818	0.783	0.750	0.778

LGBM, Light Gradient Boosting Model; Average performance from 4-fold cross validation on the training dataset and performance on the test dataset with the final model.

The microbiome features that are significant for distinguishing superagers from typical agers are detailed in Table 3. Among these features, *Alistipes* exhibited the highest importance, with a gain value of 27.14. Other notable genera with high importance scores include *PAC001137_g* (20.56), *PAC001138_g* (19.40), *Leuconostoc* (19.19), and *PAC001115_g* (15.87), each showing a gain value exceeding 10.

**Table 3:** Feature importance in the classification model for superagers.

Phylum	Class	Order	Family	Genus	Importance
Bacteroidetes	Bacteroidia	Bacteroidales	Rikenellaceae	Alistipes	27.14
Firmicutes	Clostridia	Clostridiales	Lachnospiraceae	PAC001137_g	20.56
Firmicutes	Clostridia	Clostridiales	Lachnospiraceae	PAC001138_g	19.40
Firmicutes	Bacilli	Lactobacillales	Leuconostocaceae	Leuconostoc	19.19
Firmicutes	Clostridia	Clostridiales	Christensenellaceae	PAC001115_g	15.87
Firmicutes	Clostridia	Clostridiales	Lachnospiraceae	PAC000194_g	12.77
Firmicutes	Clostridia	Clostridiales	Mogibacterium_f	PAC001236_g	11.63
Firmicutes	Clostridia	Clostridiales	Peptostreptococcaceae	Romboutsia	7.97

Higher scores of importance indicate a greater significance of the genus in contributing to the model’s ability to distinguish between superagers and typical agers.

### 3.3 SHAP results

**Figure 3:**
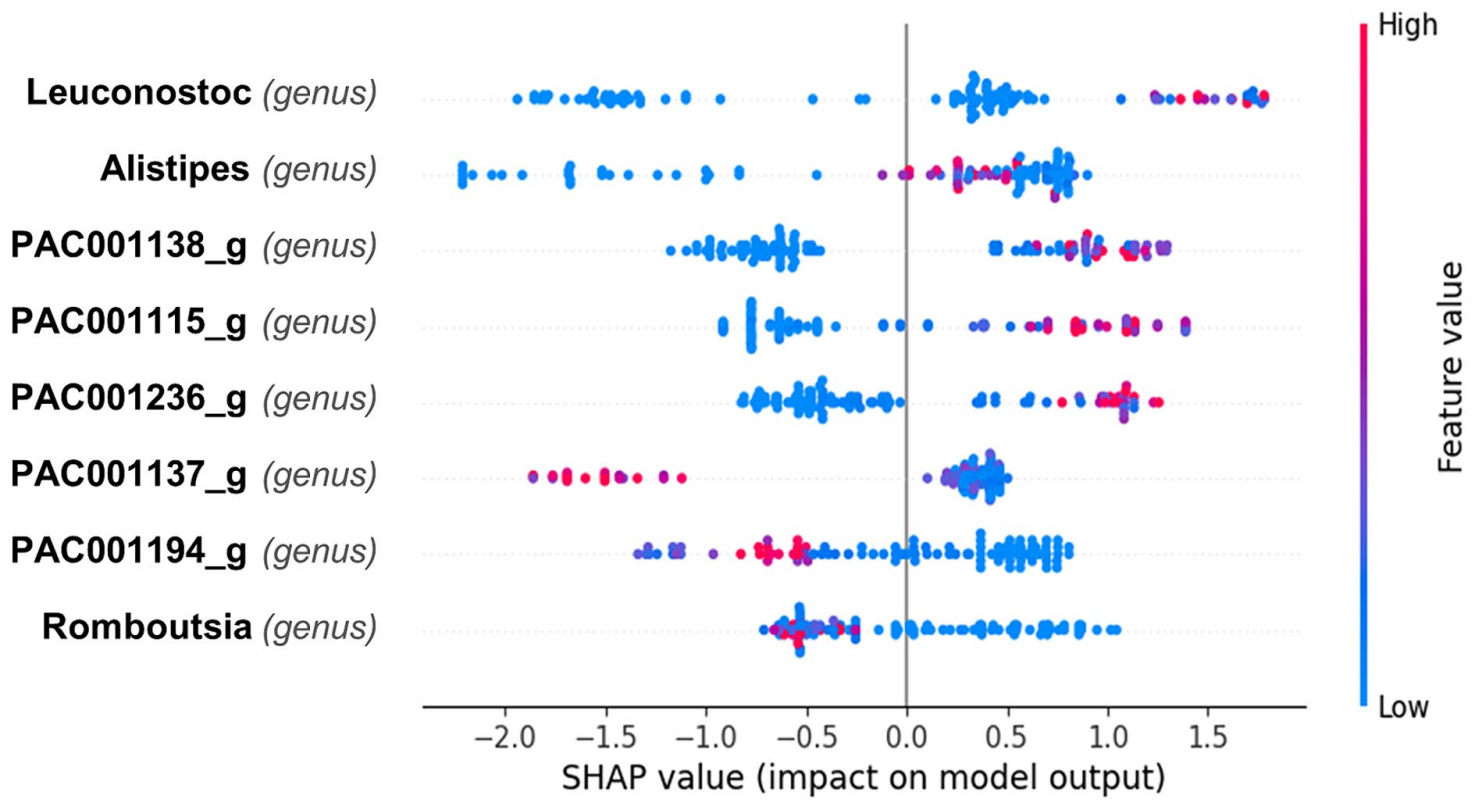
Impact of bacterial genera on predictive model for superager Classification. This Beeswarm plot aggregates SHAP values for each data point, detailing the influence of different bacterial genera on the prediction of superaging. The color of each dot indicates the abundance of the bacterial genus: red for higher and blue for lower values. Leuconostoc primarily exhibits SHAP values near zero with a slight shift toward positive values, indicating a generally neutral but potentially minor positive effect on predicting superaging when present in higher quantities. Alistipes displays a range of positive and negative SHAP values, suggesting its impact varies with the individual’s microbiome composition; however, higher abundances slightly enhance the likelihood of predicting superaging. Genera PAC001138_g, PAC001115_g, and PAC001236_g are associated with positive SHAP values at higher abundances, suggesting a positive influence on superaging predictions. Conversely, PAC001137_g, PAC001194_g, and Romboutsia are generally characterized by negative SHAP values, indicating that their greater presence may detract from predicting superaging.

The SHAP plot (Figure 3) illustrates the impact of different bacterial genera on the model’s output, with the SHAP values indicating the contribution of each feature to predicting superagers. The color gradient represents the feature values, with red indicating higher values and blue indicating lower values. Mean absolute Shapley value for each feature is presented in the Supplementary Figure 3. It reveals that Leuconostoc from Firmicutes demonstrated the highest value at 0.97, followed by Alistipes from Bacteroidetes at 0.81, and PAC001138_g from Firmicutes at 0.8.

The SHAP plot (Figure 3) presents the impact of various bacterial genera on predicting superaging. For Leuconostoc, the SHAP values are mostly clustered around zero, indicating a generally neutral effect on the prediction of superaging, although there is a slight skew toward positive values, suggesting a potential minor positive influence when present in higher quantities. Alistipes shows a mix of positive and negative SHAP values, indicating variable influences depending on the individual microbiome composition but higher values tend to have a slightly positive influence on the prediction of being a superager.

For the genera PAC001138_g, PAC001115_g, and PAC001236_g, higher abundances tend to correlate with a positive impact on the likelihood of being classified as a superager. Conversely, PAC001137_g, PAC001194_g, and Romboutsia exhibit SHAP values that are generally skewed toward the negative, indicating that higher abundances of these genera may negatively influence the prediction of superaging.

### 3.4 Correlations between probabilities of superagers and cognitive scores

The correlation analysis results, presented in Table 4, reveal significant relationships between cognitive scores and the likelihood of being classified as ‘superagers’. In the training dataset, the class probability of being classified as superagers was significantly correlated with the scores of SVLT delayed recall (*r* = 0.39, *p* < 0.001) and RCFT delayed recall (*r* = 0.54, *p* < 0.001). In contrast, in the test dataset, the class probability was correlated only with the COWAT phonemic total scores (*r* = 0.48, *p* = 0.027).

**Table 4:** Correlation results between class probability and cognitive performance.

Name	Training	Test
	Correlation (*p*-value)	Correlation (*p*-value)
Cognitive performance		
Digit span forward backward	0.088 (0.433)	0.204 (0.376)
K-BNT	0.191 (0.088)	−0.275 (0.227)
RCFT copy	0.210 (0.060)	−0.183 (0.428)
SVLT delayed recall	0.393 (< 0.001*)	0.111 (0.633)
RCFT delayed recall	0.541 (< 0.001*)	0.164 (0.478)
COWAT phonemic	0.156 (0.165)	0.481 (0.027*)
StroopTest Color reading	0.156 (0.165)	−0.049 (0.834)

BNT, Boston Naming Test; RCFT, Rey Complex Figure Test; SVLT, Seoul Verbal Learning Test; COWAT, Controlled Oral Word Association Test. All cognitive scores are shown as *z*-scores.

^*^p < 0.05, the *p* value was obtained by Spearman correlation.

## 4 Discussion

Our investigation demonstrated distinct characteristics of the gut microbiome that differentiate superagers from typical agers. Utilizing these gut microbiome features, we constructed predictive models capable of classifying superagers. In the training set, the model demonstrated a high ability to distinguish between superagers and typical agers, achieving an AUC of 0.832, indicating strong discriminatory power. Additionally, the model attained an accuracy of 0.764, meaning that 76.4% of the predictions made were correct. Notably, the model’s performance on the test dataset was robust, achieving an AUC of 0.861, which suggests that the model generalizes well to unseen data, while maintaining its discriminatory power. The accuracy on the test dataset was 0.762, closely matching the accuracy on the training dataset, highlighting the model’s reliability and stability across different datasets.

Several studies have explored classifying cognitive impairments in older adults using gut microbiome data. For example, a random forest model using bacterial data alone achieved an AUC of 0.76 for distinguishing patients with MCI from healthy controls using a trans-kingdom microbiome approach, while incorporating microbial metabolic pathways, bacteria, and viruses resulted in an AUC of 0.78 (Chaudhari et al., 2023). In another study, 12 altered genera were identified as differing between MCI and healthy control groups, with associations found with attention and executive function. A logistic regression model in this study achieved an AUC of 0.84 (Fan et al., 2023). Compared to these studies predicting cognitive dysfunction using microbiome data from healthy controls, our model exhibits advancement in predicting superagers among cognitively unimpaired older adults.

It is noteworthy that Alistipes from the Rikenellaceae family within the Bacteroidetes phylum shows the highest importance. Alistipes exhibited a combination of positive and negative SHAP values, indicating variable influences based on individual microbiome composition.

Previous studies have suggested a negative correlation between Alistipes and cognitive function (Ren et al., 2020; Muhammad et al., 2023), including associations with memory performance (Tsan et al., 2022; Jiao et al., 2023). Additionally, its abundance has been negatively correlated with the thickness of the left lateral orbitofrontal cortex, even among participants with normal cognition, subjective cognitive decline, and cognitive impairment (He et al., 2023).

However, our study showed that both high and low abundances of Alistipes had significant impact being classified as ‘superagers,’ with high abundance positively influencing prediction values. This suggests that the role of Alistipes in contributing to ‘supergaers’ may be more complex than previously anticipated. Compatible with this finding, Alistipes may have both protective effects against some diseases and pathogenic role in others (Parker et al., 2020). Some studies have correlated their presence with the promotion of healthy phenotypes, such as protective roles in conditions like colitis (Dziarski et al., 2016), autism spectrum disorder (Strati et al., 2017), and various liver (Shao et al., 2018; Sung et al., 2019) and cardiovascular disorders (Jie et al., 2017; Zuo et al., 2019). Despite these beneficial associations, Alistipes has also been shown to have pathogenic roles in diseases such as depression (Jiang et al., 2015), and colorectal cancer (Feng et al., 2015). Further studies are warranted to elucidate the role of Alistipes in cognitive function in successful aging.

We found that higher abundances of the genera PAC001138_g, PAC001115_g, and PAC001236_g, tend to correlate with a positive impact on the likelihood of being classified as superagers.

Among these, PAC001138_g, which belongs to the family Lachnospiraceae, was identified as a highly important feature. Our findings indicate that higher abundance of PAC001138_g predicts superager status. This aligns with previous research, which found that Lachnospiraceae levels were relatively reduced, particularly among oldest-old adults (Biagi et al., 2016). Given that superagers may exhibit resilience against age-associated cognitive decline, it is plausible that higher abundance of Lachnospiraceae in superagers reflects a gut-microbiome composition similar to that of younger individuals compared to typical agers. Further supporting this idea, previous research demonstrated a positive correlation between Lachnospiraceae abundance and performance on three-stage command test in patients with amnestic MCI (Liu et al., 2021). However, PAC001137_g and PAC000194_g, also from the Lachnospiraceae family, showed opposite results, indicating that these genera may negatively influence the prediction of superaging. Given the limited research related to PAC001137_g and PAC000194_g in human cognitive function, further investigation into its potential functions and impacts is necessary.

PAC001115_g, which belongs to the family Christensenellaceae, is another important feature for predicting superager status, with higher abundances correlating with a greater likelihood of being classified as a superager. This finding is consistent with existing literature that highlights the significant role of Christensenellaceae in human health. For instance, a study found that Christensenellaceae was significantly enriched in individuals with a normal BMI (18.5–24.9) compared to obese individuals (BMI ≥ 30) (Goodrich et al., 2014). Furthermore, a meta-analysis of inflammatory bowel disease, involving over 3,000 individuals, identified that Christensenellaceae as one of five taxa considered a signature of a healthy gut (Mancabelli et al., 2017). Christensenellaceae may promote gut homeostasis and healthy aging by reducing adiposity, inflammation, and the later risk for development of metabolic and cognitive dysfunction (Badal et al., 2020), its higher abundance may contribute to the cognitive health observed in superagers.

Our study found that a higher abundance of PAC001236_g, which belongs to the Mogibacterium family, positively impacts the prediction of superagers. This is in contrast to previous research where a higher abundance of Mogibacterium has been associated with negative outcomes in neurological conditions. Specifically, Park and Wu (2022) reported an increased abundance of Mogibacterium in patients with Alzheimer’s disease, and Socała et al. (2021) found similar results in individuals with schizophrenia, compared to healthy controls. Despite these findings, there is limited research exploring the relationship between PAC001236, Mogibacterium, and cognitive function in older adults. Given the contrasting roles of PAC001236_g in neurological diseases, further investigation into its potential functions and impacts is warranted.

For Leuconostoc, the SHAP values largely cluster around zero, suggesting a generally neutral influence on the prediction of superaging. However, there is a slight tendency toward positive values, indicating a potentially small positive effect when present in higher quantities. Leuconostoc is one of the most common probiotic strains that are widely used in many probiotic products. Given the numerous reported health benefits of probiotics, such as improvement of cognitive function (Ton et al., 2020) and antioxidant (Jang et al., 2018), it is plausible that a high abundance of Leuconostoc could be associated with an increased likelihood of being superagers. Similarly to our finding, Leuconostoc was less abundant in the female MCI group (Hatayama et al., 2023).

It should be also noted that memory functions, particularly those assessed by delayed recall tests of SVLT and RCFT, are strongly associated with the likelihood of being classified as superagers. Since we defined superagers clinically based on the scores of delayed recall in SVLT and RCFT, this finding suggests that our predictive model based on the gut microbiome is highly effective in distinguishing superagers from typical agers. However, in the test dataset, the correlation between the class probability and cognitive scores was only significant in the verbal fluency test assessed by COWAT phonemic test. This disparity between training and test datasets might indicate that while memory retention is a consistent predictor, verbal fluency may also be a relevant factor in different contexts or subsets of the population. Further investigation is needed to understand these relationships and their implications for identifying and supporting superagers.

Recent clinical trials using non-pharmacological intervention for cognitive function in older adults have explored whether administration of a prebiotic food supplement or nutritional support improved cognitive function. For instance, a 12-week, placebo-controlled, double blinded randomized trial of 36 twin pairs (72 individuals) aged 60 and older demonstrated that prebiotic administration resulted in a higher abundance of *Bifidobacterium* and significant improvements in cognitive function compared to the placebo group (Ni Lochlainn et al., 2024). Similarly, another study reported that a 10-week multispecies probiotic intervention led to improvements in MMSE scores, digit tasks, and depressive symptoms in healthy older adults (Ruiz-Gonzalez et al., 2024). Building on these findings, our study suggests that modulating the gut microbiome through prebiotic or probiotic interventions could be a promising approach to preserving superior cognitive function.

This research has several limitations. First, our study adopts a cross-sectional design, limiting our ability to determine whether alterations in the microbiome observed in superagers are causal factors or consequences of their cognitive status. A longitudinal study would be necessary to establish causality. Second, this study only considers the relative abundance of gut microbiome as features for the model. While relative abundance provides valuable information about the microbial composition and can easily adopted as features of model, with other microbial data, model could incorporate diverse aspects of gut microbiome. For instance, incorporating absolute bacterial counts can offer a more comprehensive view of microbial load. Additionally, to prevent the curse of dimensionality, new features can be constructed using techniques such as Principal Component Analysis (PCA), which reduce the complexity of the data while preserving sufficient information for prediction. Furthermore, including additional data types, such as dietary intakes and demographic information. Third, in addressing missing values, we selected a subset of bacteria with fewer missing values. While this approach aimed to mitigate the impact of missing data, it may have inadvertently excluded potentially meaningful features that could distinguish between superagers and typical agers. Alternatives such as replacing missing values using statistical methods like mean, median, max, and min or employing machine learning regression models to predict and fill in missing values could be considered to handle this issue more effectively. Additionally, our study is constrained by the use of only LightGBM models. Although these models have demonstrated good performance across various studies (Yanagawa et al., 2024; Sun et al., 2020), exploring a wider range of machine learning algorithms or ensemble methods could uncover additional insights and potentially lead to better performance. Moreover, if more data were available, deep learning algorithms could be adapted for improved performance. Finally, while SHAP values from estimations are desirable according to the original paper (Lundberg and Lee, 2017), there are still some drawbacks. For instance, KernelSHAP ignores feature dependence, which may lead to errors. On the other hand, TreeSHAP does not suffer from this issue; however, it can yield non-intuitive feature importance values (Linardatos et al., 2020). Additionally, while SHAP enhances interpretability, it may come at the cost of reduced accuracy (Vimbi et al., 2024). Considering these challenges, we utilized SHAP solely for understanding the directional impact of features, without regarding SHAP values as indicative of feature importance in this study. Finally, although recent studies have shown a link between the apolipoprotein E *(APOE)* genotype and gut microbiome composition (Hammond et al., 2023) —specifically, that individuals carrying the *APOE* ε4 allele tend to have higher levels of pro-inflammatory microbes—, we did not include *APOE* ε4 carrier status in our model. Future studies could consider incorporating *APOE* ε4 carrier status to further explore its potential impact on gut microbiome composition and cognitive health.

Despite these limitations, this study is the first to identify unique patterns of the gut microbiome in superagers and to develop machine learning-based predictive models that can differentiate superagers from typical agers with reasonably high performance. These findings pave the way for future research to explore the relationships between gut microbiome composition and cognitive health.

Our newly developed model has significant potential for practical application, particularly in clinical settings. It could be integrated into diagnostic that assesses an individual’s likelihood of being a superager based on their gut microbiome profile, along with other key features such as cognitive scores and lifestyle factors. Such a tool could help identify individuals who are more resilient to cognitive decline, enabling personalized interventions that target gut health to support healthy cognitive aging. Moreover, identifying specific microbial features associated with cognitive resilience could guide the development of targeted probiotic or dietary interventions aimed at promoting cognitive longevity.

To validate these findings, longitudinal studies are essential to confirm the relationship between the gut microbiome and cognitive resilience. Additionally, incorporating a broader range of microbial data and exploring various machine learning algorithms could enhance the predictive accuracy and robustness of these models. This approach could offer deeper insights into the role of the gut microbiome in aging and cognitive function, ultimately guiding the development of more effective interventions for promoting cognitive health in older adults.

## Data availability statement

The datasets presented in this article are not readily available because the datasets used for these analyses are available from the corresponding author upon reasonable request. However, it is important to note that the data are not available for public access or to third parties other than the original researchers of this study, in accordance with participant consent agreements. Requests to access the datasets should be directed to Geon Ha Kim, geonha@ewha.ac.kr.

## Ethics statement

The studies involving humans were approved by Institutional Review Board of Ewha Womans University Mokdong Hospital. The studies were conducted in accordance with the local legislation and institutional requirements. The participants provided their written informed consent to participate in this study.

## Author contributions

HK: Conceptualization, Formal analysis, Investigation, Writing – original draft, Methodology, Validation, Visualization, Writing – review & editing. BK: Investigation, Data curation, Writing – review & editing. SH: Data curation, Methodology, Writing – review & editing. SS: Data curation, Methodology, Writing – review & editing. JJ: Conceptualization, Data curation, Writing – review & editing. GK: Conceptualization, Data curation, Formal analysis, Funding acquisition, Investigation, Supervision, Writing – original draft, Writing – review & editing.

## Footnotes

^1^ https://www.ezbiocloud.net/contents/16smtp

## References

[ref1] Badal V. D. Vaccariello E. D. Murray E. R. Yu K. E. Knight R. Jeste D. V.. (2020). The gut microbiome, aging, and longevity: a systematic review. Nutrients 12:3759. doi: 10.3390/nu12123759, PMID: 33297486 PMC7762384

[ref2] Baek M. J. Kim K. Park Y. H. Kim S. (2016). The validity and reliability of the Mini-mental state Examination-2 for detecting mild cognitive impairment and Alzheimer’s disease in a Korean population. PLoS One 11:e0163792. doi: 10.1371/journal.pone.0163792, PMID: 27668883 PMC5036810

[ref3] Biagi E. Franceschi C. Rampelli S. Severgnini M. Ostan R. Turroni S.. (2016). Gut microbiota and extreme longevity. Curr. Biol. 26, 1480–1485. doi: 10.1016/j.cub.2016.04.016 27185560

[ref4] Bott N. T. Bettcher B. M. Yokoyama J. S. Frazier D. T. Wynn M. Karydas A.. (2017). Youthful processing speed in older adults: genetic, biological, and behavioral predictors of cognitive processing speed trajectories in aging. Front. Aging Neurosci. 9:55. doi: 10.3389/fnagi.2017.00055, PMID: 28344553 PMC5344896

[ref5] Chaudhari D. S. Jain S. Yata V. K. Mishra S. P. Kumar A. Fraser A.. (2023). Unique trans-kingdom microbiome structural and functional signatures predict cognitive decline in older adults. Geroscience 45, 2819–2834. doi: 10.1007/s11357-023-00799-1, PMID: 37213047 PMC10643725

[ref6] Dang C. Harrington K. D. Lim Y. Y. Ames D. Hassenstab J. Laws S. M.. (2019). Superior memory reduces 8-year risk of mild cognitive impairment and dementia but not amyloid beta-associated cognitive decline in older adults. Arch. Clin. Neuropsychol. 34, 585–598. doi: 10.1093/arclin/acy078, PMID: 30272115

[ref7] Depp C. A. Jeste D. V. (2006). Definitions and predictors of successful aging: a comprehensive review of larger quantitative studies. Am. J. Geriatr. Psychiatry 14, 6–20. doi: 10.1097/01.JGP.0000192501.03069.bc, PMID: 16407577

[ref8] Dziarski R. Park S. Y. Kashyap D. R. Dowd S. E. Gupta D. (2016). Pglyrp-regulated gut microflora *Prevotella falsenii*, Parabacteroides distasonis and *Bacteroides eggerthii* enhance and *Alistipes finegoldii* attenuates colitis in mice. PLoS One 11:e0146162. doi: 10.1371/journal.pone.0146162 26727498 PMC4699708

[ref9] Fan K. C. Lin C. C. Liu Y. C. Chao Y. P. Lai Y. J. Chiu Y. L.. (2023). Altered gut microbiota in older adults with mild cognitive impairment: a case-control study. Front. Aging Neurosci. 15:1162057. doi: 10.3389/fnagi.2023.1162057 37346147 PMC10281289

[ref10] Feng Q. Liang S. Jia H. Stadlmayr A. Tang L. Lan Z.. (2015). Gut microbiome development along the colorectal adenoma-carcinoma sequence. Nat. Commun. 6:6528. doi: 10.1038/ncomms7528, PMID: 25758642

[ref11] Galland L. (2014). The gut microbiome and the brain. J. Med. Food 17, 1261–1272. doi: 10.1089/jmf.2014.7000, PMID: 25402818 PMC4259177

[ref12] Gefen T. Peterson M. Papastefan S. T. Martersteck A. Whitney K. Rademaker A.. (2015). Morphometric and histologic substrates of cingulate integrity in elders with exceptional memory capacity. J. Neurosci. 35, 1781–1791. doi: 10.1523/JNEUROSCI.2998-14.2015, PMID: 25632151 PMC4308613

[ref13] Goodrich J. K. Waters J. L. Poole A. C. Sutter J. L. Koren O. Blekhman R.. (2014). Human genetics shape the gut microbiome. Cell 159, 789–799. doi: 10.1016/j.cell.2014.09.053 25417156 PMC4255478

[ref14] Hammond T. C. Green S. J. Jacobs Y. Chlipala G. E. Xing X. Heil S.. (2023). Gut microbiome association with brain imaging markers, APOE genotype, calcium and vegetable intakes, and obesity in healthy aging adults. Front. Aging Neurosci. 15:1227203. doi: 10.3389/fnagi.2023.1227203, PMID: 37736325 PMC10510313

[ref15] Harrison T. M. Maass A. Baker S. L. Jagust W. J. (2018). Brain morphology, cognition, and β-amyloid in older adults with superior memory performance. Neurobiol. Aging 67, 162–170. doi: 10.1016/j.neurobiolaging.2018.03.024 29665578 PMC5955827

[ref16] Harrison T. M. Weintraub S. Mesulam M.-M. Rogalski E. (2012). Superior memory and higher cortical volumes in unusually successful cognitive aging. J. Int. Neuropsychol. Soc. 18, 1081–1085. doi: 10.1017/S1355617712000847 23158231 PMC3547607

[ref17] Hatayama K. Ebara A. Okuma K. Tokuno H. Hasuko K. Masuyama H.. (2023). Characteristics of intestinal microbiota in Japanese patients with mild cognitive impairment and a risk-estimating method for the disorder. Biomedicines 11:1789. doi: 10.3390/biomedicines11071789, PMID: 37509429 PMC10376419

[ref18] He B. Sheng C. Yu X. Zhang L. Chen F. Han Y. (2023). Alterations of gut microbiota are associated with brain structural changes in the spectrum of Alzheimer’s disease: the SILCODE study in Hainan cohort. Front. Aging Neurosci. 15:1216509. doi: 10.3389/fnagi.2023.1216509 37520126 PMC10375500

[ref19] Hedden T. Gabrieli J. D. (2004). Insights into the ageing mind: a view from cognitive neuroscience. Nat. Rev. Neurosci. 5, 87–96. doi: 10.1038/nrn1323 14735112

[ref20] Jang H. J. Song M. W. Lee N.-K. Paik H.-D. (2018). Antioxidant effects of live and heat-killed probiotic *Lactobacillus plantarum* Ln1 isolated from kimchi. J. Food Sci. Technol. 55, 3174–3180. doi: 10.1007/s13197-018-3245-4 30065428 PMC6045990

[ref21] Jiang H. Ling Z. Zhang Y. Mao H. Ma Z. Yin Y.. (2015). Altered fecal microbiota composition in patients with major depressive disorder. Brain Behav. Immun. 48, 186–194. doi: 10.1016/j.bbi.2015.03.016, PMID: 25882912

[ref22] Jiao Y. Zhao Z. Li X. Li L. Xiao D. Wan S.. (2023). Salidroside ameliorates memory impairment following long-term ethanol intake in rats by modulating the altered intestinal microbiota content and hippocampal gene expression. Front. Microbiol. 14:1172936. doi: 10.3389/fmicb.2023.1172936, PMID: 37362918 PMC10288325

[ref23] Jie Z. Xia H. Zhong S.-L. Feng Q. Li S. Liang S.. (2017). The gut microbiome in atherosclerotic cardiovascular disease. Nat. Commun. 8:845. doi: 10.1038/s41467-017-00900-1, PMID: 29018189 PMC5635030

[ref24] Komanduri M. Gondalia S. Scholey A. Stough C. (2019). The microbiome and cognitive aging: a review of mechanisms. Psychopharmacology 236, 1559–1571. doi: 10.1007/s00213-019-05231-1 31055629

[ref25] Linardatos P. Papastefanopoulos V. Kotsiantis S. (2020). Explainable AI: a review of machine learning interpretability methods. Entropy 23:18. doi: 10.3390/e23010018, PMID: 33375658 PMC7824368

[ref26] Liu P. Jia X. Z. Chen Y. Yu Y. Zhang K. Lin Y. J.. (2021). Gut microbiota interacts with intrinsic brain activity of patients with amnestic mild cognitive impairment. CNS Neurosci. Ther. 27, 163–173. doi: 10.1111/cns.13451, PMID: 32929861 PMC7816203

[ref27] Lundberg S. M. Lee S. I. (2017). A unified approach to interpreting model predictions. Adv Neur In:30. doi: 10.48550/arXiv.1705.07874

[ref28] Mancabelli L. Milani C. Lugli G. A. Turroni F. Cocconi D. van Sinderen D.. (2017). Identification of universal gut microbial biomarkers of common human intestinal diseases by meta-analysis. FEMS Microbiol. Ecol. 93:fix153. doi: 10.1093/femsec/fix153, PMID: 29126267

[ref29] Martin C. R. Osadchiy V. Kalani A. Mayer E. A. (2018). The brain-gut-microbiome Axis. Cell. Mol. Gastroenterol. Hepatol. 6, 133–148. doi: 10.1016/j.jcmgh.2018.04.003, PMID: 30023410 PMC6047317

[ref30] Mohajeri M. H. Fata G. L. Steinert R. E. Weber P. (2018). Relationship between the gut microbiome and brain function. Nutr. Rev. 76, 481–496. doi: 10.1093/nutrit/nuy009 29701810

[ref31] Muhammad J. A. Ngouongo Y. J. W. Ramirez S. Kautz T. F. Satizabal C. L. Himali J. J.. (2023). Poor cognition is associated with increased abundance of Alistipes and decreased abundance of Clostridium genera in the gut. Alzheimers Dement. 19:e076520. doi: 10.1002/alz.076520

[ref32] Ni Lochlainn M. Bowyer R. C. E. Moll J. M. Garcia M. P. Wadge S. Baleanu A. F.. (2024). Effect of gut microbiome modulation on muscle function and cognition: the PROMOTe randomised controlled trial. Nat. Commun. 15:1859. doi: 10.1038/s41467-024-46116-y, PMID: 38424099 PMC10904794

[ref33] Park S. Wu X. (2022). Modulation of the gut microbiota in memory impairment and Alzheimer’s disease via the inhibition of the parasympathetic nervous system. Int. J. Mol. Sci. 23:13574. doi: 10.3390/ijms232113574, PMID: 36362360 PMC9657043

[ref34] Parker B. J. Wearsch P. A. Veloo A. C. M. Rodriguez-Palacios A. (2020). The genus Alistipes: gut Bacteria with emerging implications to inflammation, Cancer, and mental health. Front. Immunol. 11:906. doi: 10.3389/fimmu.2020.00906 32582143 PMC7296073

[ref35] Pedregosa F. Varoquaux G. Gramfort A. Michel V. Thirion B. Grisel O.. (2011). Scikit-learn: machine learning in Python. J. Mac. Learn. Res. 12, 2825–2830. doi: 10.48550/arXiv.1201.0490

[ref36] Reichstadt J. Depp C. A. Palinkas L. A. Jeste D. V. (2007). Building blocks of successful aging: a focus group study of older adults’ perceived contributors to successful aging. Am. J. Geriatr. Psychiatry 15, 194–201. doi: 10.1097/JGP.0b013e318030255f 17322132

[ref37] Ren T. Gao Y. Qiu Y. Jiang S. Zhang Q. Zhang J.. (2020). Gut microbiota altered in mild cognitive impairment compared with Normal cognition in sporadic Parkinson’s disease. Front. Neurol. 11:137. doi: 10.3389/fneur.2020.00137, PMID: 32161568 PMC7052381

[ref38] Rogalski E. J. Gefen T. Shi J. Samimi M. Bigio E. Weintraub S.. (2013). Youthful memory capacity in old brains: anatomic and genetic clues from the northwestern SuperAging project. J. Cogn. Neurosci. 25, 29–36. doi: 10.1162/jocn_a_00300, PMID: 23198888 PMC3541673

[ref39] Ruiz-Gonzalez C. Cardona D. Rueda-Ruzafa L. Rodriguez-Arrastia M. Ropero-Padilla C. Roman P. (2024). Cognitive and emotional effect of a multi-species probiotic containing lactobacillus rhamnosus and *Bifidobacterium lactis* in healthy older adults: a double-blind randomized placebo-controlled crossover trial. Probiotics Antimicrob. Proteins. doi: 10.1007/s12602-024-10315-2, PMID: 38935259 PMC12532666

[ref40] Ryu H. J. Yang D. W. (2023). The Seoul neuropsychological screening battery (SNSB) for comprehensive neuropsychological assessment. Dement. Neurocogn. Disord. 22, 1–15. doi: 10.12779/dnd.2023.22.1.1, PMID: 36814700 PMC9939572

[ref41] Shao L. Ling Z. Chen D. Liu Y. Yang F. Li L. (2018). Disorganized gut microbiome contributed to liver cirrhosis progression: a Meta-omics-based study. Front. Microbiol. 9:3166. doi: 10.3389/fmicb.2018.03166 30631318 PMC6315199

[ref42] Socała K. Doboszewska U. Szopa A. Serefko A. Włodarczyk M. Zielińska A.. (2021). The role of microbiota-gut-brain axis in neuropsychiatric and neurological disorders. Pharmacol. Res. 172:105840. doi: 10.1016/j.phrs.2021.105840 34450312

[ref43] Stadlbauer V. Engertsberger L. Komarova I. Feldbacher N. Leber B. Pichler G.. (2020). Dysbiosis, gut barrier dysfunction and inflammation in dementia: a pilot study. BMC Geriatr. 20, 248–213. doi: 10.1186/s12877-020-01644-2, PMID: 32690030 PMC7372911

[ref44] Strati F. Cavalieri D. Albanese D. De Felice C. Donati C. Hayek J.. (2017). New evidences on the altered gut microbiota in autism spectrum disorders. Microbiome 5:24. doi: 10.1186/s40168-017-0242-1, PMID: 28222761 PMC5320696

[ref45] Sun F. W. Stepanovic M. R. Andreano J. Barrett L. F. Touroutoglou A. Dickerson B. C. (2016). Youthful brains in older adults: preserved neuroanatomy in the default mode and salience networks contributes to youthful memory in superaging. J. Neurosci. 36, 9659–9668. doi: 10.1523/JNEUROSCI.1492-16.2016 27629716 PMC5039247

[ref46] Sun X. Liu M. Sima Z. (2020). A novel cryptocurrency price trend forecasting model based on LightGBM. Financ. Res. Lett. 32:101084. doi: 10.1016/j.frl.2018.12.032

[ref47] Sung C. M. Lin Y. F. Chen K. F. Ke H. M. Huang H. Y. Gong Y. N.. (2019). Predicting clinical outcomes of cirrhosis patients with hepatic encephalopathy from the fecal microbiome. Cell. Mol. Gastroenterol. Hepatol. 8, 301–318.e2. doi: 10.1016/j.jcmgh.2019.04.008, PMID: 31004827 PMC6718362

[ref48] Ton A. M. M. Campagnaro B. P. Alves G. A. Aires R. Côco L. Z. Arpini C. M.. (2020). Oxidative stress and dementia in Alzheimer’s patients: effects of synbiotic supplementation. Oxidative Med. Cell. Longev. 2020, 1–14. doi: 10.1155/2020/2638703, PMID: 32411323 PMC7201593

[ref49] Tsan L. Sun S. Hayes A. M. R. Bridi L. Chirala L. S. Noble E. E.. (2022). Early life Western diet-induced memory impairments and gut microbiome changes in female rats are long-lasting despite healthy dietary intervention. Nutr. Neurosci. 25, 2490–2506. doi: 10.1080/1028415X.2021.1980697 34565305 PMC8957635

[ref50] Vimbi V. Shaffi N. Mahmud M. (2024). Interpreting artificial intelligence models: a systematic review on the application of LIME and SHAP in Alzheimer’s disease detection. Brain Inform 11:10. doi: 10.1186/s40708-024-00222-1 38578524 PMC10997568

[ref51] Virtanen P. Gommers R. Oliphant T. E. Haberland M. Reddy T. Cournapeau D.. (2020). SciPy 1.0: fundamental algorithms for scientific computing in Python. Nat. Methods 17, 261–272. doi: 10.1038/s41592-019-0686-2, PMID: 32015543 PMC7056644

[ref52] Yanagawa R. Iwadoh K. Akabane M. Imaoka Y. Bozhilov K. K. Melcher M. L.. (2024). LightGBM outperforms other machine learning techniques in predicting graft failure after liver transplantation: creation of a predictive model through large-scale analysis. Clin. Transpl. 38:e15316. doi: 10.1111/ctr.15316, PMID: 38607291

[ref53] Zhang J. Andreano J. M. Dickerson B. C. Touroutoglou A. Barrett L. F. (2020). Stronger functional connectivity in the default mode and salience networks is associated with youthful memory in superaging. Cereb. Cortex 30, 72–84. doi: 10.1093/cercor/bhz071 31058917 PMC7029690

[ref54] Zuo K. Li J. Li K. Hu C. Gao Y. Chen M.. (2019). Disordered gut microbiota and alterations in metabolic patterns are associated with atrial fibrillation. Gigascience 8:giz058. doi: 10.1093/gigascience/giz058, PMID: 31149718 PMC6543127

