# Supplementary material for: Predicting superagers: a machine learning approach utilizing gut microbiome features
Source: Front Aging Neurosci. 2024 Sep 9;16:1444998. doi: 10.3389/fnagi.2024.1444998 (PMC11417495; doi:10.3389/fnagi.2024.1444998)
Supplement: Supplementary file 1 [file Table_1.DOCX]

***Supplementary Material***

| 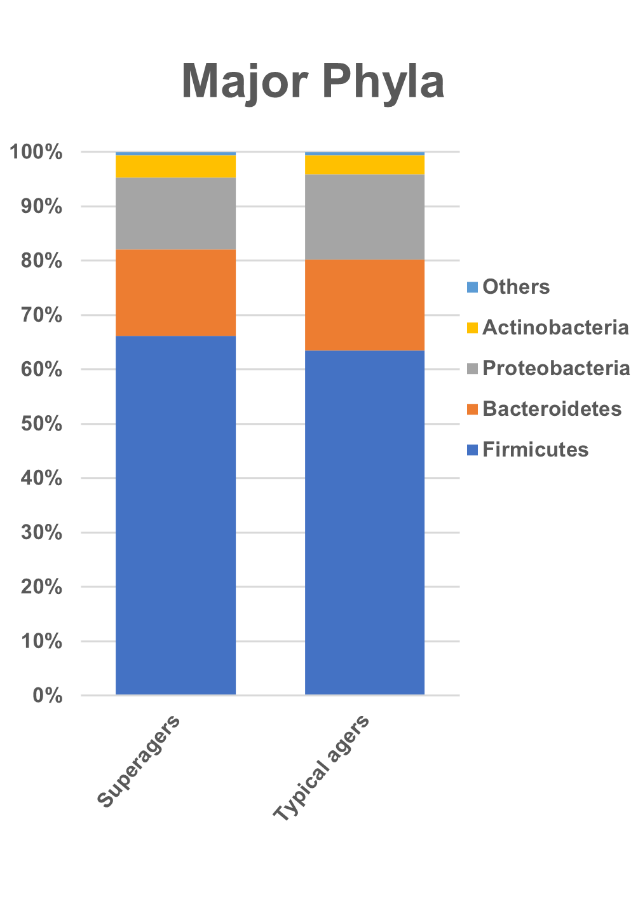 | 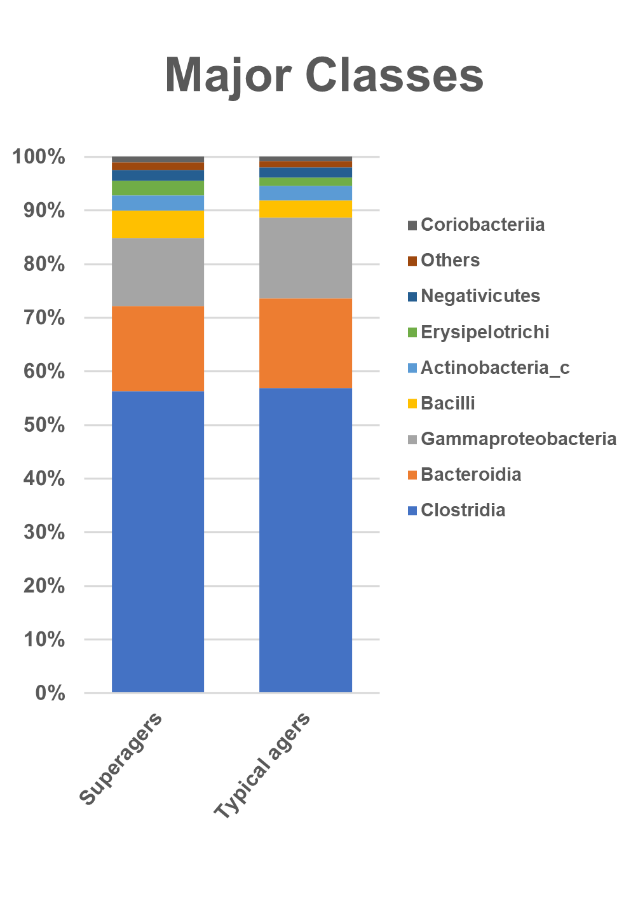 |
| --- | --- |
| **Supplementary Figure 1. Relative abundance of phyla and classes between superagers and typical agers.** For relative abundance of phyla and classes between superagers and typical agers, no differences were exhibited. | |

| **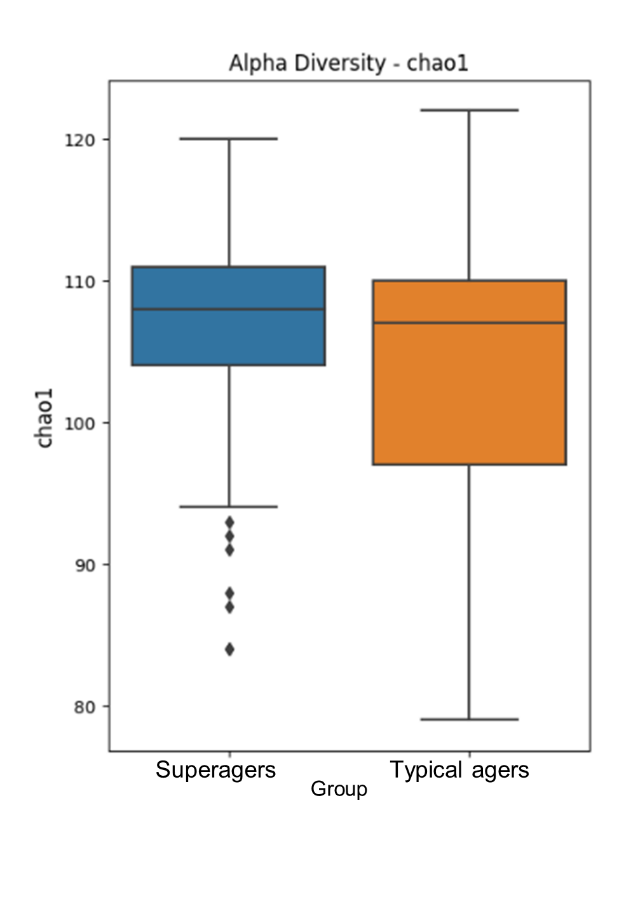** | **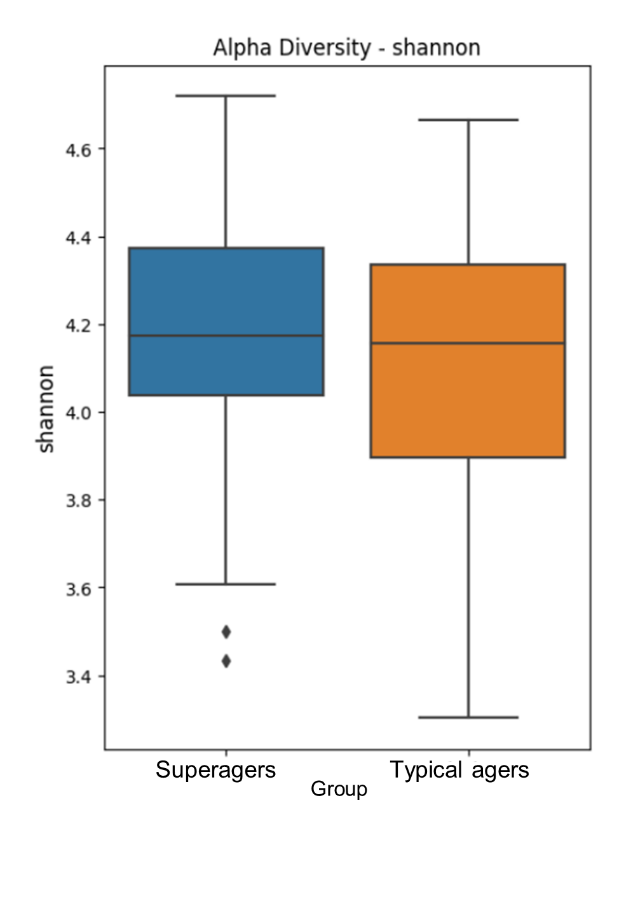** |
| --- | --- |
| **Supplementary Figure 1. Box-plots of alpha diversity between super agers and typical agers.** For both alpha diversity and beta diversity, no differences were shown between superagers and typical agers | |


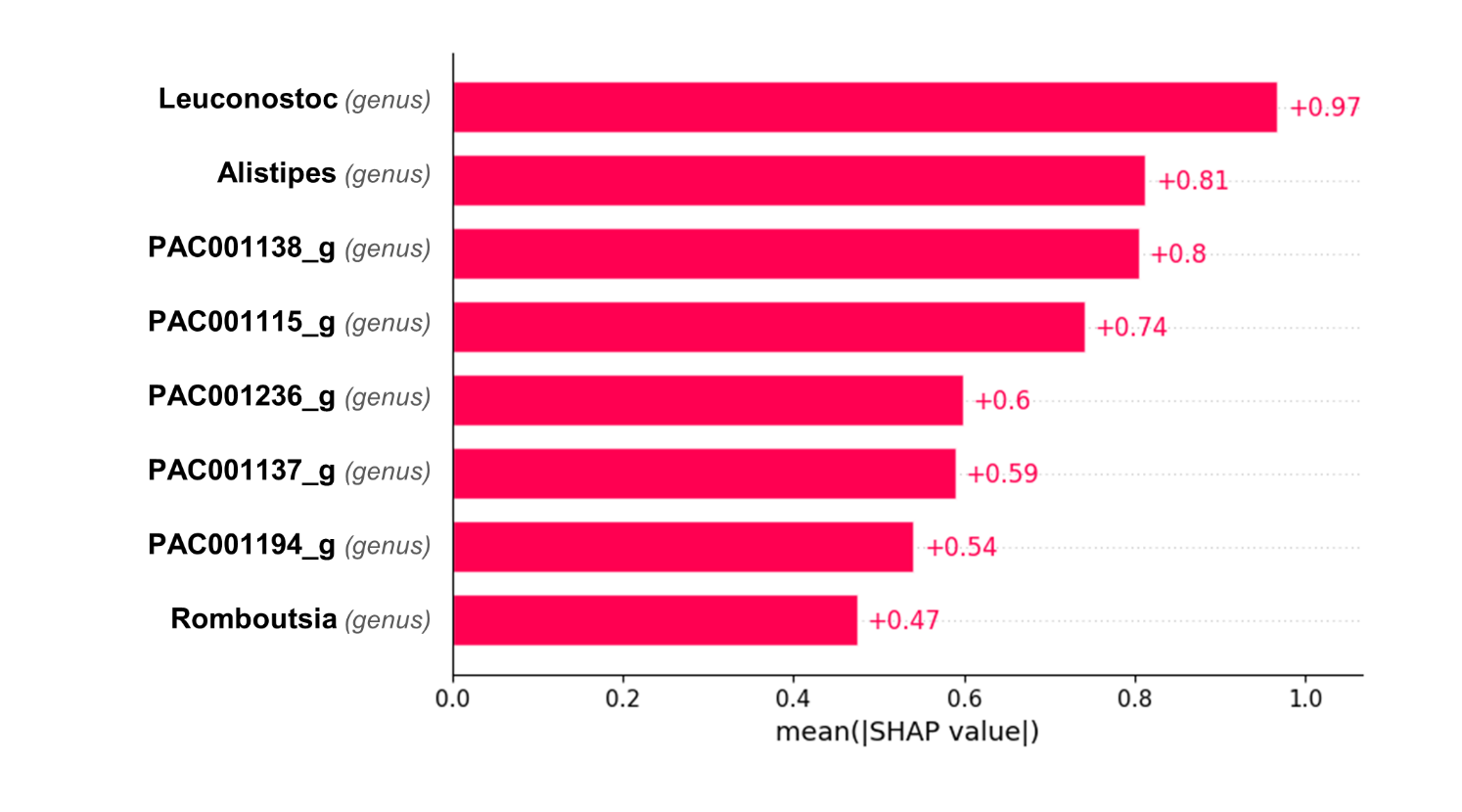


**Supplementary Figure 2. Mean absolute Shapley value for each feature**. Leuconostoc from Firmicutes demonstrated the highest value at 0.97, followed by Alistipes from Bacteroidetes at 0.81, and PAC001138_g from Firmicutes at 0.8.
